# Association Between Clinical Measures of Depth of Sedation and Multimodal Cerebral Physiology in Acute Traumatic Neural Injury

**DOI:** 10.1089/neur.2024.0090

**Published:** 2024-10-02

**Authors:** Kangyun Park, Logan Froese, Tobias Bergmann, Alwyn Gomez, Amanjyot Singh Sainbhi, Nuray Vakitbilir, Abrar Islam, Kevin Y. Stein, Izzy Marquez, Fiorella Amenta, Younis Ibrahim, Frederick A. Zeiler

**Affiliations:** ^1^ Undergraduate Medicine, Rady Faculty of Health Sciences, University of Manitoba, Winnipeg, Canada.; ^2^ Biomedical Engineering, Faculty of Engineering, University of Manitoba, Winnipeg, Canada.; ^3^ Biosystems Engineering, Faculty of Engineering, University of Manitoba, Winnipeg, Canada.; ^4^ Section of Neurosurgery, Department of Surgery, Rady Faculty of Health Sciences, University of Manitoba, Winnipeg, Canada.; ^5^ Department of Human Anatomy and Cell Science, Rady Faculty of Health Sciences, University of Manitoba, Winnipeg, Canada.; ^6^ Centre on Aging, University of Manitoba, Winnipeg, Canada.; ^7^ Division of Anaesthesia, Department of Medicine, Addenbrooke’s Hospital, University of Cambridge, Cambridge, United Kingdom.; ^8^ Department of Clinical Neuroscience, Karolinska Institutet, Stockholm, Sweden.

**Keywords:** Bispectral Index, Cerebral Autoregulation, Critical Care, Depth of Sedation, Intracranial Pressure, Pressure Reactivity Index, Richmond Agitation Sedation Scale, Traumatic Brain Injury, RASS

## Abstract

Neurointensive care primarily focuses on secondary injury reduction, utilizing a variety of guideline-based approaches (including administration of high-dose sedation) to reduce the injured state. However, titration of sedation is currently based on the Richmond Agitation Sedation Scale (RASS), a subjective clinical grading score of a patient’s response to external physical stimuli, and not an objective measure. Therefore, it is likely that there exists substantial variation in objective sedation depth for a given clinical grade in these patients, leading to undesired sedation depths and cerebral physiological consequences. Improper sedation can impede cerebral autoregulation, emphasizing the critical need for optimal sedation in traumatic brain injury (TBI) patients. This study evaluates the relationship between RASS to an objective measure of depth of sedation (bispectral index, BIS) and cerebral physiological measures. Fifty-nine patients were assessed using Jonckheere–Terpstra testing to compare various key physiologies with RASS. RASS (−5 through 0 categories) showed no statistically significant relationship between BIS and cerebral physiological parameters, after adjusting for multiple comparisons. Furthermore, it is crucial to note that within each RASS value, the distribution of the physiological measures all had high variability. As an exemplar, for RASS values of −5 and −4, BIS ranged from near 0 (burst suppression levels) up to over 80 (near awake states). BIS and other cerebral physiologies displayed substantial variation across each RASS category. This suggests that RASS as a means to titrate sedative medication for the goal of neuroprotection is insufficient. More momentary, individualized determination of sedation depth is required for TBI patients.

## Background

Traumatic brain injuries (TBIs), related to acute biomechanical neural trauma, are a leading cause of death in the first 40 years of life globally.^1^ Not only that, TBIs are a huge financial burden to the global economy (∼400 billion USD/year).^2^ Secondary injuries occurring after moderate/severe TBIs drive poor long-term outcomes.^2^ The secondary injuries can take the form of metabolic derangements, neuroinflammation, progressive edema, and impairment of blood flow/oxygen/nutrient delivery.^3^ Naturally, the goal in the intensive care unit (ICU) is minimizing and preventing secondary injury. However, current TBI care is limited by the nonindividualized approach to care, with recent work linking more personalized targets to improved outcomes.^3–7^

Currently, the level of sedation is most often measured clinically by the Richmond Agitation Sedation Scale (RASS).^8,9^ RASS is a subjective clinical grade that is done at the bedside, easy to use, intuitive, and cheap for health care professionals.^8,9^ Despite the universal use of RASS, it has many limitations. RASS is not suitable for patients with impairments such as visual, auditory, or somatosensory due to their respective deficits. There is a known interpatient heterogenetic dose–response to different sedative agents that is further complicated by the patient’s comorbidities, which RASS cannot account for.^10–13^ Furthermore, different sedatives affect different individuals variably on cerebral blood flow regulation and metabolism in both healthy and diseased states.^10,13^ Thus, once the patient is heavily sedated, RASS becomes less useful and cannot be used to reliably predict a reduction in metabolic demand,^8,9^ which raises questions about the clinical utility of RASS as a depth of sedation measure. Within the same clinically determined RASS value, the depth of sedation and the neuronal activity may vary greatly given the limited assessment of neural and metabolic activity.

Bispectral index (BIS) is a level of sedation scoring system that uses electroencephalography entropy to quantify the level of sedation from 0 to 100 arbitrary units (au).^14^ BIS is a well-established method of determining the depth of sedation in operative settings and it offers an objective quantification of dose–response between drugs administered and the patient’s level of sedation,^14,15^ in hopes to better quantify how much sedatives the patients legitimately require. Historically, the relationship between RASS and BIS has been varied in strength.^15^ Moreover, BIS has recently been linked to other measures of cerebrovascular function to determine an optimal level of sedation with a patient, which in theory offers more neuroprotection from pressure-passive flow states.^16,17^ Such precision of sedation depth cannot be attained with RASS and calls into question the spectrum of cerebral physiological insult that the TBI patients are exposed to across the range of RASS values. As a result, the purpose of this observational study is to evaluate the link between RASS and BIS/other continuous cerebral physiologies. This will lay the groundwork for the clinical relevance and accuracy of RASS as a means to titrate sedation for secondary injury prevention/reduction in acute TBI care.

## Methods

### Patient population

Data was retrospectively extracted from the Winnipeg Acute TBI Database^18^ including adult TBI patient data from January 2019 to March 2023. All adult patients who were admitted to the surgical intensive care unit at the Health Sciences Centre Winnipeg (Manitoba, Canada) with moderate/severe TBI for invasive cerebral physiological monitoring had their data collected as a part of the ongoing Winnipeg Acute TBI Database. Treatments conformed to the Brain Trauma Foundation (BTF) guidelines.^19^ Patients were monitored continuously for RASS, BIS left (BIS_L), BIS right (BIS_R), intracranial pressure (ICP), and mean arterial pressure (MAP). Of note, BIS was not used by the treating team to titrate sedation or dictate clinical care provision for any patients included in this study.

### Ethical approval

Ethics for all aspects of data collection has been obtained (H2017:181, H2017:188), with approval for access to the database for physiological analysis (H2020:118; B2019:065; B2023:001).

### Data collection

Basic patient demographics, injury patterns, treatments, drug information, and serially measured RASS scores were collected. Clinical RASS scores and drug information was directly collected and digitized from the bedside nursing charts with date/time markups recorded. During the patient’s ICU stay, all patients underwent invasive ICP and arterial blood pressure (ABP) monitoring, as per BTF guidelines.^19^ ICP monitoring used an intraparenchymal strain gauge probe (Codman ICP MicroSensor; Codman & Shurtlef Inc., Raynham, MA) placed in the frontal lobe and for ABP, radial arterial lines connected to pressure transducers (Baxter Healthcare Corp. CardioVascular Group, Irvine, CA). ICP and ABP were sampled at a frequency of 100 Hz from patients’ bedside monitors using Intensive Care Monitoring “Plus” (ICM+) (Cambridge Enterprise Ltd, Cambridge, UK, http://icmplus.neurosurg.cam.ac.uk), through an analog-to-digital signal conversion (DT9804/DT9826, Data Translations, Marlboro, MA).

Similarly, all patients (*n* = 59) included in this study had BIS and at least one channel of near-infrared spectroscopy (NIRS) regional cerebral oxygen saturation (rSO_2_) recorded during their ICU stay. Of note, 55 patients also had rSO_2_ recorded from both their left and right sides. There was one patient with only rSO_2_ on the left side and three patients with only rSO_2_ on the right side. Recordings of rSO_2_ were achieved using NIRS regional oximetry of the left and right frontal lobes (Covidien INVOS 5100C https://www.medtronic.com/covidien/en-us/products/cerebral-somatic-oximetry/invos-5100c-cerebral-somatic-oximeter.html). BIS was recorded bilaterally using the Covidien BIS Complete 4-Channel Monitor (Medtronic of Canada Ltd., Brampton, ON, Canada, www.metronic.com/covidien).

Signal artifacts were removed using both manual and automated methods. For BIS signals, this also included the evaluation of EMG signals linked to BIS for erroneous activity and other factors that can interfere with BIS viability. This archived high-frequency digital physiological data was then used to derive measures of cerebral blood flow autoregulation, brain compliance, and cerebral perfusion pressure (CPP). Of note, none of the patients included in this study was diagnosed with seizures or epileptic activity during the course of their recordings.

### Signal processing

Signal processing work was done with data processed in ICM+ or R statistical software (R Core Team (2019). R: A language and environment for statistical computing [R Foundation for Statistical Computing (version 2023.03.1 + 446), Vienna, Austria. URL https://www.R-project.org/]. ABP and ICP were decimated over a 10-sec nonoverlapping moving average filter to get MAP and ICP, focusing on the frequency range associated with cerebral vasomotion.^20,21^ CPP was derived as MAP–ICP.

Three separate ICP-based metrics of cerebrovascular reactivity (surrogate for pressure autoregulation) were derived and include the following: A. pressure reactivity index (PRx), B. pulse amplitude index (PAx), and C. the correlation between pulse amplitude of ICP (AMP) and CPP (i.e., RAC index). PRx, PAx, and RAC are all alternative measures of cerebrovascular reactivity ranging from −1 to 1.^22–27^ Past work has shown that higher values indicate more impaired cerebrovascular reactivity, and lower values indicate intact cerebrovascular reactivity.^27–30^ PRx was derived as a Pearson correlation between 30 consecutive 10-sec windows of ICP and MAP, updated every minute.^22,23,31^ For the derivation of PAx and RAC, AMP was derived using Fourier analysis of the ICP pulse waveform.^24,32^ PAx was then derived as the correlation between the slow waves of AMP and MAP,^24,32^ and RAC was derived as the correlation between the slow waves of AMP and CPP.^25^ The compensatory reserve index (RAP) was then derived as the moving correlation between the AMP and ICP.^33–36^

### Statistical analyses

R Statistical computing software was used to conduct all statistical analyses. The following packages were used within the R Statistical Computing software: *ggplot2*, *clinfun*, *coin*, *ggpubr*. Alpha for all described testing was set at 0.05 initially. With correction for multiple comparisons using the Bonferroni correction methodology, the alpha was set to 0.0083. This adjustment was made due to 6 comparisons of RASS (−5 to 0). All continuous cerebral physiological data was found to be nonparametric in nature. Basic descriptive statistics were used to summarize the population, using mean, median, standard deviation, interquartile range (IQR), and raw counts, for patient demographics where appropriate.

**FIG. 1.**
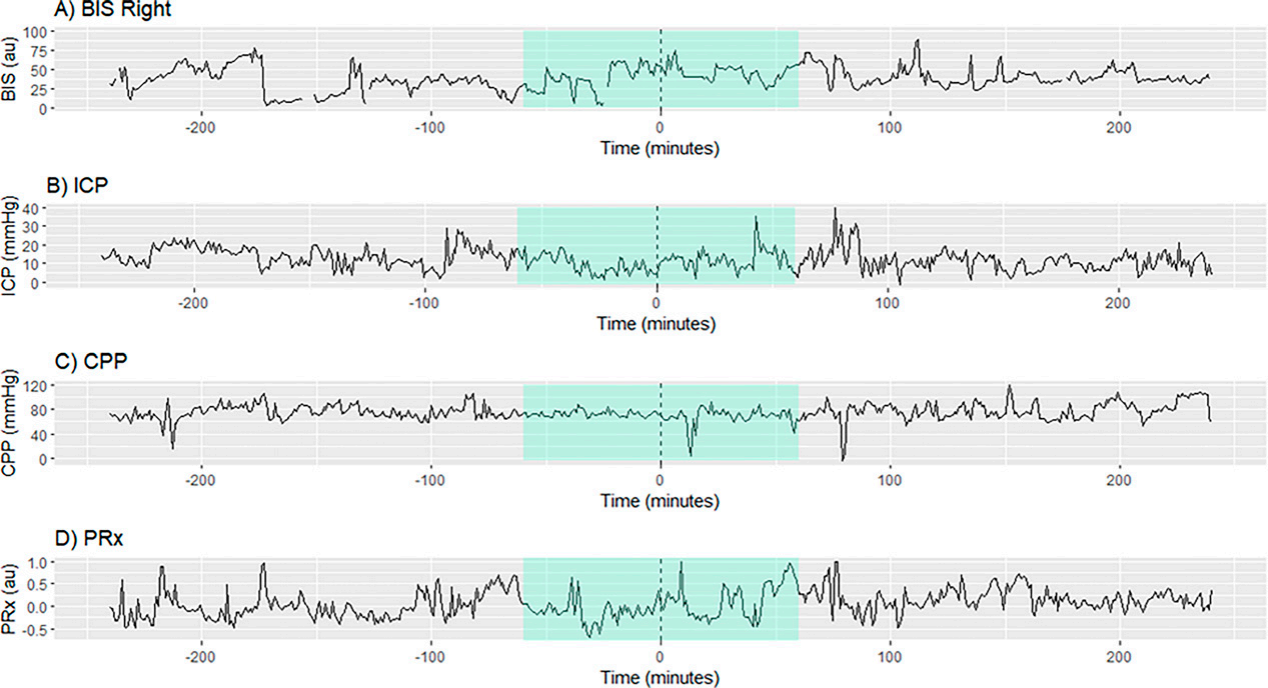
Example of Windowing method for cerebral physiology around RASS measures. Figure 1 is an example to visualize how the data was managed, and how the time intervals were created. The dotted line in the figure at time 0 min depicts when the RASS reading was recorded at the bedside by a health care professional. The highlighted portion is where RASS is assumed to be constant (60 min before and after the RASS reading). Thus, the highlighted portion makes up 120 min, creating our 120-min window of time. BIS, bispectral index; ICP, intracranial pressure; CPP, cerebral perfusion pressure; PRx, pressure reactivity index; Au, arbitrary units; mmHg, millimeters of mercury.

Windows of time were used to estimate the preceding and subsequent RASS values before the bedside recording; and this methodology can be visualized in Figure 1 (with 60/30/15 min before and after the RASS value used to create a 120/60/30-min windows of time). We then compared median and variance in BIS signals within and between RASS scores (−5 and 0; RASS scores from −5 [comatose/unrousable] to 0 [alert and calm]). Similarly, we evaluated the variance in pressure-flow dynamics (ICP, MAP, and CPP), cerebrovascular reactivity/autoregulation (PRx, PAx, and RAC), compensatory reserve (RAP), and oxygen delivery (rSO_2_), within and between different RASS scores and BIS, evaluating median and variance.

Finally, we compared RASS and % time above key literature-defined thresholds. Thus, the following cerebral physiological measures were evaluated in relation to RASS:

1. BIS mean left and right.
2. ICP mean and % time with ICP above 20 and 22.5 mmHg extracted from the BTF guidelines.^33^
3. CPP mean and % time with CPP below 60 mmHg and above 70 mmHg extracted from the BTF guidelines.^3,28^
4. PRx mean and % time with PRx above 0, +0.25, and +0.35 from literature-defined thresholds.^28,29,34^
5. PAx mean and % time with PAx above 0 and +0.25 from literature-defined thresholds.^29,34^
6. RAC mean and % time with RAC above −0.10, −0.05, and 0 from literature-defined thresholds.^29,34^
7. RAP mean and % time with RAP above +0.40 and 0.^35–37^
8. rSO_2_ mean and % time with rSO_2_ below 50%/60%/70%/80%/90%.^30,38^

Histograms were created to assess data distribution at each RASS score value for the population, repeated for BIS (left and right) (Appendix 1), mean ICP, mean CPP, mean PRx, mean PAx, mean RAC, mean RAP, and mean rSO_2_ (right) (Appendix 2). Mean rSO_2_ left and right sides were the same and had no significant differences. Boxplots of each physiological measure were plotted against different values of RASS (Appendix 3) and BIS (Appendix 4). Each boxplot was subjected to a Jonckheere–Terpstra test to statistically assess median values across RASS scores and BIS. Of note, for the above-described analysis approach, each window length variation for physiology around RASS scoring and BIS performed nearly identically. Thus, all figures and data reported in the results have been created using the 120-min window around each RASS score or minute by minute BIS values (for the 120-min RASS window).

Finally, a subgroup analysis was performed focusing on the primary sedative agents used at our facility (propofol, fentanyl, and midazolam) and the impact on BIS, to comment on the utility of weight-adjusted dosing in prediction of cerebral physiological responses with sedation escalation. This work compared the main sedation dosing values to BIS, with other physiologies and drug relationships found in our previous work.^11,12,39,40^ To complete this work, both boxplots and contour plots were used to demonstrate the relationships between these factors.

## Results

### Patient demographics

From the Winnipeg Acute TBI Database, 59 patients were included in the study. The median age was 41 (IQR: 27.5–59.5) and 81.4% were male. All patients had a moderate/severe TBI with a median Glasgow Coma Scale (GCS) score of 6 (IQR: 4–8) with the median calculated arterial partial pressure of CO_2_ at 37 mmHg (IQR: 34–40). A total of 189, 249, 35, 14, 5, 6 RASS scorings were seen in the population for RASS values of −5, −4, −3, −2, −1, and 0, respectively. A summary of the patient demographics can be found in Table 1.

**Table 1.** Patient Demographics of the Winnipeg TBI Patients Included in the Study

	Median (IQR) or number (%)
Number of patients	59
Age (years)	41.0 (27.5–59.5)
Sex	
Male	48 (81.4%)
Female	11 (18.6%)
GCS	6 (4–7.5)
GCS motor	4 (2–5)
Pupil reactivity	
Bilateral reactive	38 (64.4%)
Bilateral unreactive	8 (13.6%)
Unilateral unreactive	13 (22.0%)
Hypoxia (Yes)	17 (28.8%)
Hypotension (Yes)	9 (15.3%)
Marshall CT score (admission)	
II	3 (5.1%)
III	17 (28.8%)
IV	10 (16.9%)
V	29 (49.2%)
Epidural hematoma (Yes)	5 (8.5%)
Arterial pH	7.43 (7.39–7.47)
Arterial pCO_2_	37 (34–40)
Arterial pO_2_	109 (87–138)
Propofol patients	55 (93%)
Propofol dose (mg/kg/min)	4 (3–5)
Fentanyl patients	36 (61%)
Fentanyl dose (µg/kg/min)	200 (100–300)
Midazolam patients	14 (23%)
Midazolam dose (µg/kg/h)	20 (10–20)

GCS, Glasgow Coma Scale; IQR, interquartile range; h, hour; mg, milligram; min, minute; pH, potential of hydrogen; pCO_2_, partial pressure of carbon dioxide; pO_2_, partial pressure of oxygen; µg, microgram.

### RASS and BIS relationship analysis

**FIG. 2.**
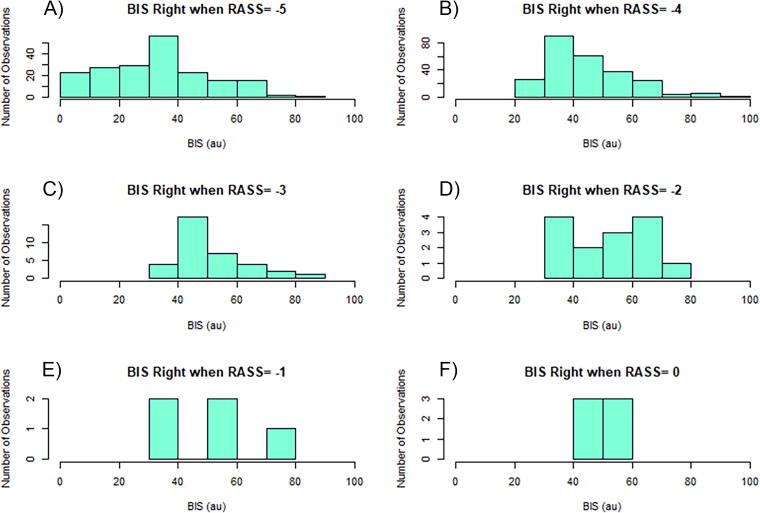
Histograms for when RASS is −5 to 0, for BIS right at the 120-min time frame. Panel A is a histogram of BIS_R when RASS is −5. Panel B is a histogram of BIS_R when RASS is −4. Panel C is a histogram of BIS_R when RASS is −3. Panel D is a histogram of BIS_R when RASS is −2. Panel E is a histogram of BIS_R when RASS is −1. Panel F is a histogram of BIS_R when RASS is 0. RASS, Richmond Agitation Sedation Scale; BIS, bispectral index; au, arbitrary unit.

BIS_R demonstrated substantial variance and distribution for every RASS value evaluated from −5 to 0 (histograms Fig. 2) and showed a wide range of values spanning across almost all the possible BIS values ranging from 0 to 100 when RASS is −5 to −3 (RASS values that are important in neuroprotection-based sedation strategies commonly used in the ICU). BIS_R values for RASS of −5, −4, −3, −2, −1, and 0 were 33.90 (IQR: 19.85–43.42), 40.77 (IQR: 35.12–51.41), 48.99 (IQR: 43.51–54.65), 50.395 (IQR: 40.425–66.333), 50.06 (IQR: 35.22–52.94), and 49.975 (IQR: 48.06–52.805), respectively. BIS_R values of ∼40 au for all RASS scores were the most prevalent in Figure 2A and 2B. BIS_L showed essentially the same histograms as BIS_R (Appendix 1).

**FIG. 3.**
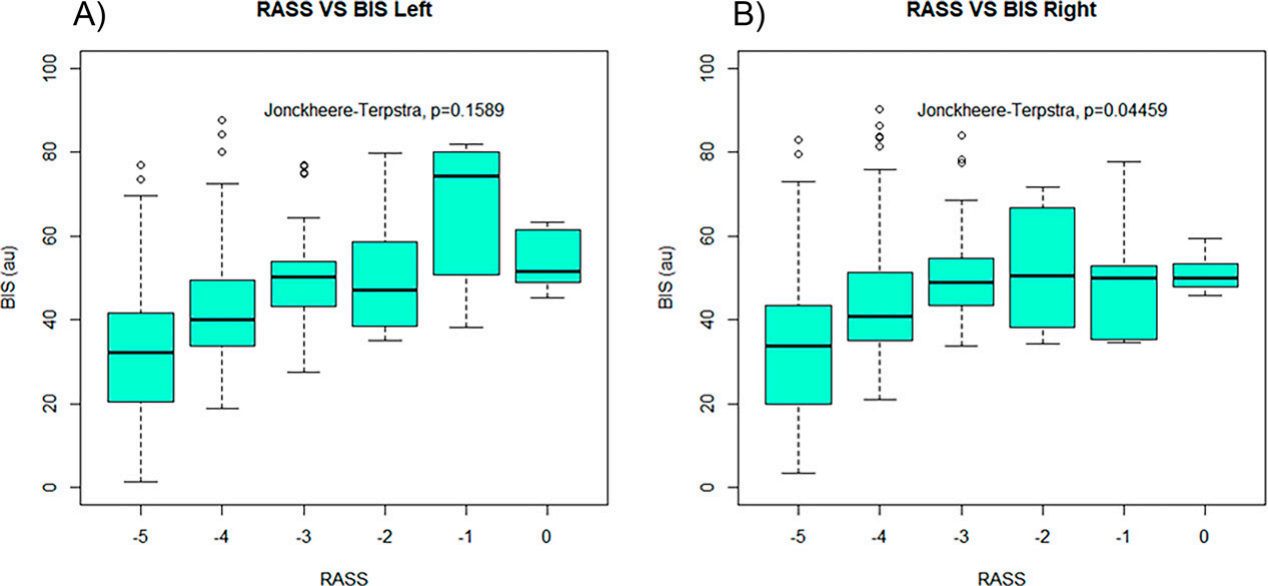
Boxplots of RASS versus BIS left and BIS right at the 120-min time frame. Panel A is a boxplot of BIS_L for RASS values −5 to 0. Panel B is a boxplot of BIS_R for RASS values −5 to 0. RASS, Richmond Agitation Sedation Scale; BIS, bispectral index; au, arbitrary units.

Panels A and B in Figure 3 show no statistically significant association between RASS and BIS as sedation increases from 0 to at RASS of −5 (*p* = 0.1589 and 0.0446), although there is a trend toward decreased BIS values between RASS of −3 and −5 (*p* < 0.005 on this subanalysis). It must be acknowledged, our number of observations for RASS values greater than −3 is very limited, and thus, it is difficult to make any conclusions looking at RASS values of −2 to 0. This guides us back to RASS values of −5 to −3, which have demonstrated extreme variation in objective sedation depth (i.e., BIS) for a given RASS clinical score (Fig. 2, Fig. 3, Appendix 1, and Appendix 2).

### RASS and cerebral physiological relationship analysis

**FIG. 4.**
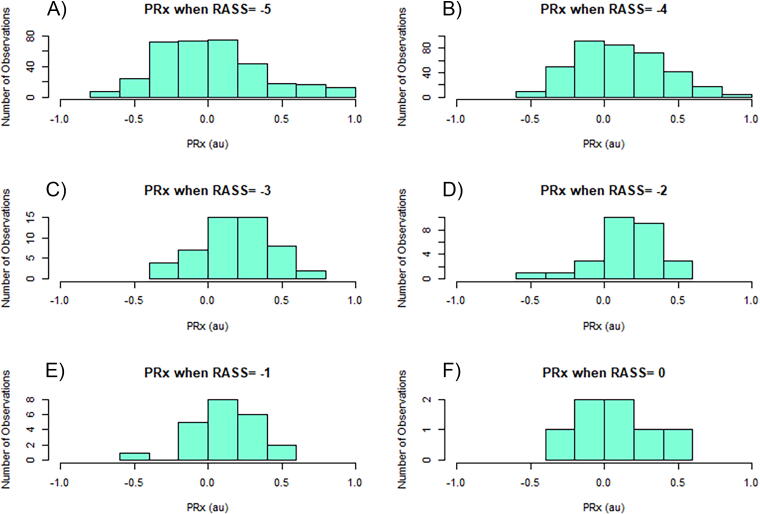
Histograms of PRx with RASS scores of −5 to 0. Panel A is a histogram of PRx when RASS is −5. Panel B is a histogram of PRx when RASS is −4. Panel C is a histogram of PRx when RASS is −3. Panel D is a histogram of PRx when RASS is −2. Panel E is a histogram of PRx when RASS is −1. Panel F is a histogram of PRx when RASS is 0. PRx, pressure reactivity index; RASS, Richmond Agitation Sedation Scale; au, arbitrary units.

There was limited association between different aspects of cerebral physiology and specific RASS values, with significant variation in physiological values for each RASS score. This is highlighted within the histograms for each cerebral physiological variable at each RASS value. Figure 4 provides an example of this.

**FIG. 5.**
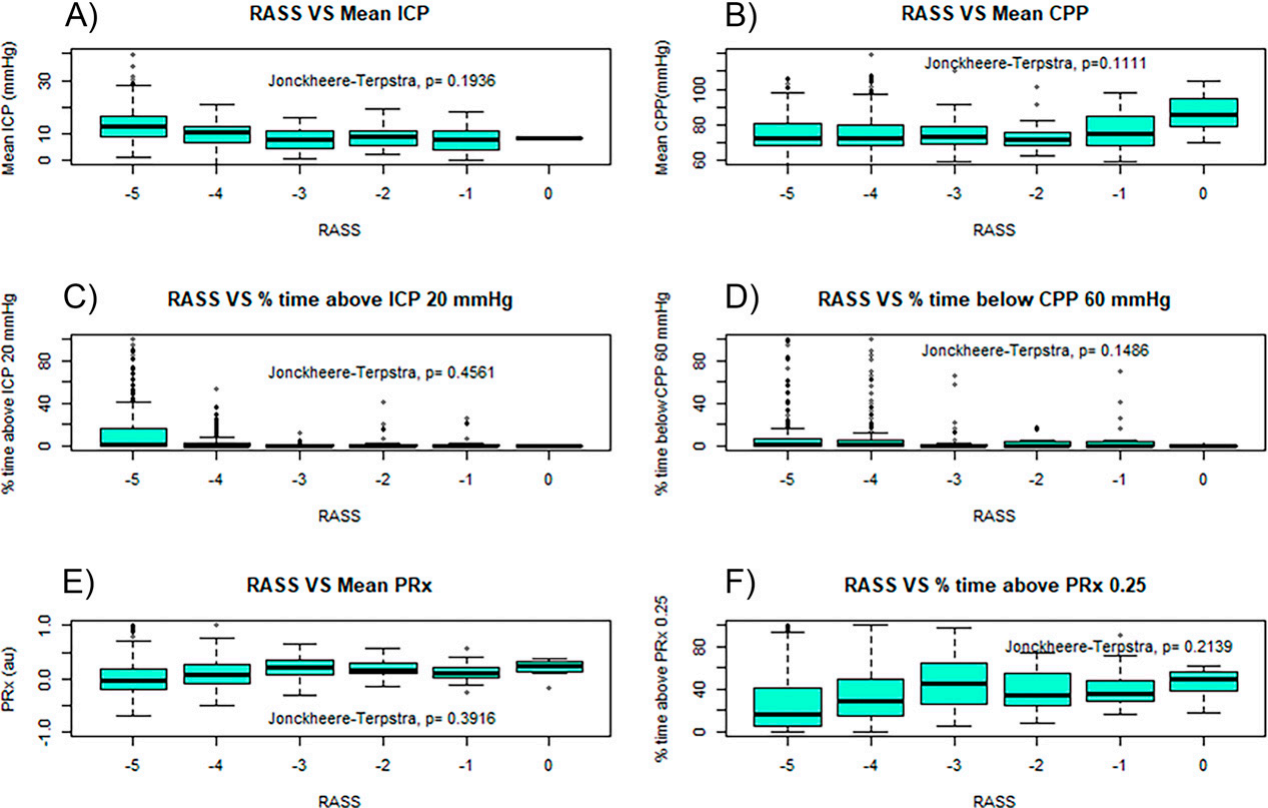
RASS versus mean ICP, CPP, and PRx thresholds in the 120-min time frame. Panel A is a boxplot of mean ICP for RASS values −5 to 0. Panel B is a boxplot of mean CPP for RASS values −5 to 0. Panel C is a boxplot of % time above ICP 20 mmHg for RASS values −5 to 0. Panel D is a boxplot of % time below CPP 60 mmHg for RASS values −5 to 0. Panel E is a boxplot of mean PRx for RASS values −5 to 0. Panel F is a boxplot of % time above PRx 0.25 for RASS values −5 to 0. RASS, Richmond Agitation Sedation Scale; ICP, intracranial pressure; CPP, cerebral perfusion pressure; PRx, pressure reactivity index; au, arbitrary unit; mmHg, millimeters of mercury.

Evaluating for difference in cerebral physiology between RASS scores, all but RAP failed to demonstrate a statistically significant relationship. For all measures, RASS seems to lose association if its value is out of the −5 to −3 range (i.e., where the majority of our data fell). Of note, however, the IQR in the boxplots for all cerebral physiological measures is overlapping in virtually all boxplots despite a visually appearing trend between these metrics and a progressive decrease in RASS. Looking at Figure 5A, the mean ICP had no relationship when compared with RASS (*p* = 0.194). There was no observable association or statistical significance with mean CPP (Fig. 5B) from different RASS values (*p* = 0.111). Figure 5 (panels C through F, respectively) displays the boxplots of RASS versus % time above ICP 20 mmHg, % time below CPP 60 mmHg, mean PRx, and % time above PRx 0.25. PRx is currently the gold standard for cerebrovascular reactivity monitoring^41^ and fails to show a relationship with RASS, with no significant differences noted between RASS scores (*p* = 0.392). Notably, the relationship between cerebrovascular reactivity and RASS showed no significant association for % time over the threshold for PRx, PAx, and RAC (*p* > 0.005, see Appendix 3). RAP was the only metric that demonstrated a significant association difference between RASS scores (*p* < 0.005 for all RAP metrics). For other Jonckheere–Terpstra testing, refer to Appendix 3.

### BIS and cerebral physiological relationship analysis

There was no association between different BIS values and aspects of cerebral physiology for this cohort (as seen in Appendix 4). In all comparisons the Jonckheere–Terpstra test failed to reach significance.

### Weight-adjusted sedative dosing and sedation depth

**FIG. 6.**
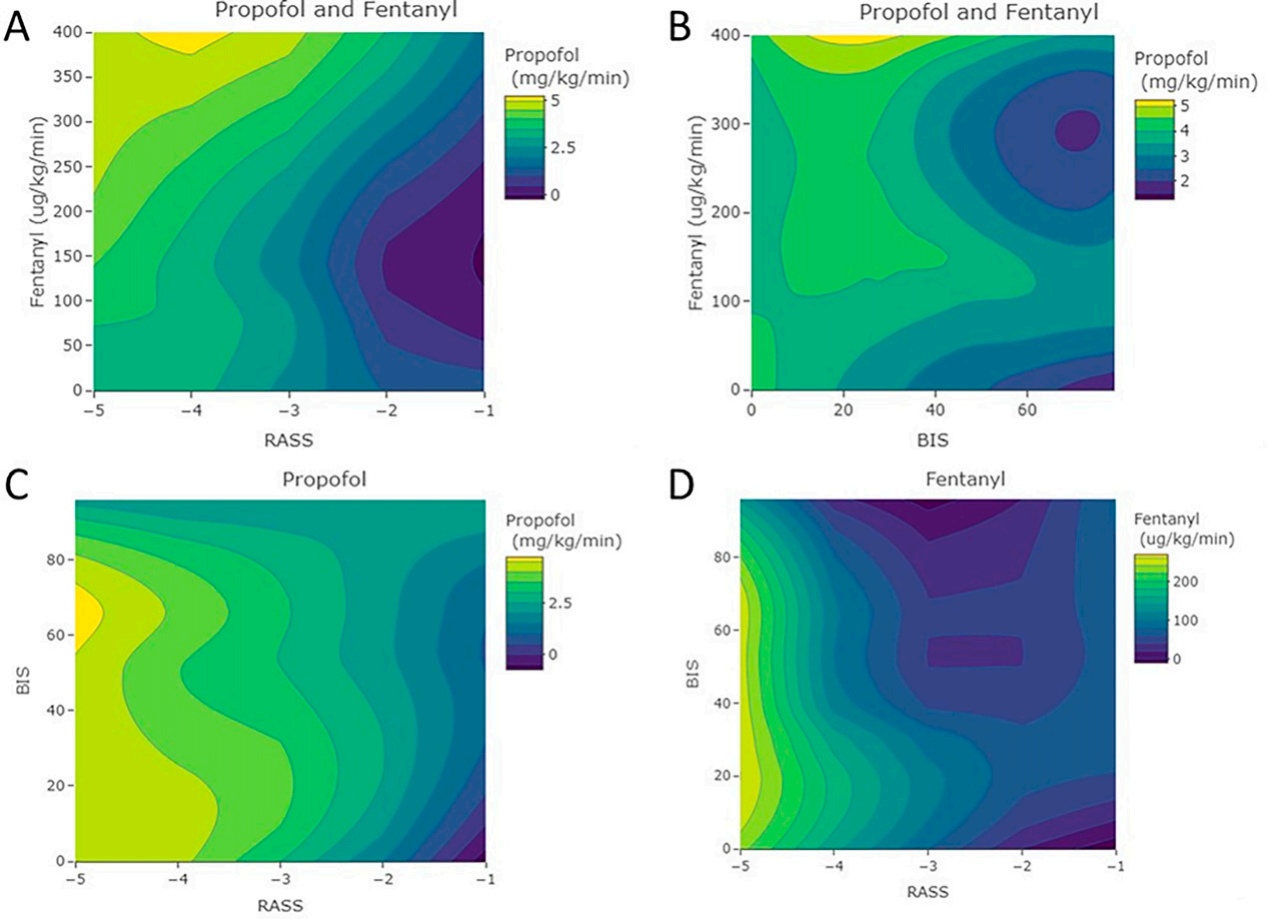
Contour plots of drug/sedation relationship. Panels show the contour plots that demonstrate the heat map relationship between propofol, fentanyl, BIS, and RASS. Panel A is propofol, fentanyl, and RASS, Panel B is propofol, fentanyl, and BIS, Panel C is propofol, BIS, and RASS, and Panel D is fentanyl, BIS, and RASS. RASS, Richmond Agitation Sedation Scale; h, hour; kg, kilogram; mg, milligram; min, minute; BIS, bispectral index; µg, microgram.

All subanalyses based on propofol, fentanyl, or midazolam weight-adjusted dosing can be found in Appendix 5 and Figure 6. Propofol and fentanyl are the primary sedative agents used at our facility to induce sedation (*n* = 55 and *n* = 36, respectively). From this analysis, although limited in statistical power, we see that propofol, fentanyl, and midazolam demonstrate no significant relationship with objectively measured BIS. This suggests that, similar to RASS scoring, weight-adjusted dosing values of sedative agents are not a reliable predictor of cerebral physiological responses.

## Discussion

RASS has been a standard scoring system to quantify agitation and sedation, and is a low-cost and fast way to measure a patient’s sedation.^8^ However, given the importance of proper depth of sedation in TBI patients for the purpose of neuroprotection, exploration into the true association between RASS and cerebral physiology was required. The work conducted in this study provides a preliminary, but an important, insight into the utility of RASS-targeted sedation management in acute TBI patients. Overall, we have demonstrated significant variability in objective BIS sedation levels and cerebral pressure-flow dynamics for a given RASS value. This suggests that RASS may have a limited utility as a means to target sedation for the purpose of neuroprotection.

More specifically, the results showed that there is no statistically significant relationship between RASS and objectively measured depth of sedation (through BIS) in moderate/severe TBI patients for RASS of −5 through 0. There is some significance when evaluating the subgroup of RASS values of −5 to −3, values that pertain to high sedation states for a patient. However, the histogram of RASS versus BIS demonstrates that −5, −4, and −3 have similar distributions (with similar medians and IQRs). Thus, RASS as a very crude overall measure of gross patient population has some value, but not as the momentary individualized determination of the depth of sedation. Furthermore, there is a high variation in the RASS versus BIS histogram from under 20 (burst suppression levels) up to over 80 (near awake states), which means that clinically there are wide variations in what is being determined at the bedside for these values. The wide IQR can be seen to overlap in virtually all boxplots suggesting significant data variance and distribution for every cerebral physiological measure at each RASS value. This indicates that RASS poorly predicts objective sedation state (as emphasized by the BIS analysis) and cannot be relied upon to assume good secondary injury protection (as emphasized by the wide array of cerebral physiology insults seen at each RASS value).

Furthermore, similar to the BIS and RASS relationship, RASS with other cerebral measures such as PRx, PAx, and RAC failed to show a significant relationship for RASS of −5 through 0. There was some general trend toward a decrease in median values when just evaluating RASS values of −3 through −5. However, given the nature of the distribution (i.e., similar overall ranges and values) and the fact that significantly impaired patients will often have −5 RASS (heavily sedated), this is to be expected. Histograms in Appendix 2 for all of these physiological measures also had a substantial variation within each RASS value and span almost all of the possible physiological values (i.e., PRx spans from −0.75 to 1 when RASS is −5). Therefore, taken with the BIS associations, although RASS as a crude clinical value provides some insight, for the emerging area of individual momentary care focused on neuroprotection, RASS is grossly inadequate to titrate sedation requirements.

This is further compounded by the limited association with RASS and systemic physiologies such as CPP and MAP. Past work has linked systemic pressure as a driving factor associated with cerebrovascular reactivity as well as other intracranial measures,^26,42,43^ with more recent and robust time-series modeling of such data documenting time-series links.^39,40,44,45^ Recent interest within critical care TBI has endeavored to explore the relationship and potential mediation of more individualized patient care. RASS determined on a person-by-person basis with limited high-resolution capabilities means that it cannot be used for such an individualized method of assessment. With recent work potentially optimizing cerebrovascular reactivity with both systemic blood pressure and sedation depth means that the clinical importance of RASS may be limited.^17,46,47^ Thus, the need to move away from clinical sedation depth scoring in TBI care is required and an objective measure of neuronal function should be adopted. Clinically, minimizing secondary injuries is key to a good patient outcome, and proper depth of sedation is very crucial to achieve this.^4^

Next, BIS (an objective measure of sedation) and the compared cerebral physiological variables did not have a relationship within the gross cohort comparisons. However, this is likely due to the variation within patient sedation depth response and patient care. Moreover, this work focused on evaluating ordinal categories of BIS and their gross median changes of specific cerebral physiological variables to mirror the RASS analysis. More advanced time-series methods need to be applied to truly understand the momentary responses of cerebral pressure-flow physiology to changes in BIS. In other work that we have completed, using this population to evaluate nonlinear high-frequency relationships, we have documented a unique individualized patient relationship between BIS (objective sedation) and PRx (cerebrovascular reactivity).^16,17^ Unlike RASS, BIS is a high-frequency measure, more dynamic to changing patient states, and thus, combined with other dynamic measures such as PRx or RAP, offers a more personalized and responsive measure of patient state. This emphasizes that RASS as a clinical measure is insufficient to measure objective sedation.

Finally, as the subgroup analysis based on sedative agents demonstrates, there was no relationship with recorded BIS values. This was the case for propofol, fentanyl, and midazolam, suggesting that the weight-adjusted dosing values provide little insight into the objective cerebral physiological response. This supports the need for continuous objective sedation depth monitoring in this population.

## Limitations/Future Directions

This pilot study explores the relationship between RASS and more objective measures. Despite the findings, more research is required to further validate these findings. First, our overall patient population was low at 59. RASS values from −2 to 0 had less than 15 BIS observations, and thus, it is difficult to draw any meaningful relationships to any objective measures of sedation past this RASS value. With more data and patients, more definitive relationships and conclusions can be drawn. Furthermore, all patients in our study are from a single-center database, presumably with a different population base than other locations. Another limitation of using RASS in our study is that the amount and frequency of RASS values are dictated by the health care provider, leading to irregular observation and limited quantities at times. Our assumption of RASS being unchanged within the 120-min time frame could be a limitation, but no significant differences were observed between 120-, 60-, and 30-min time frames. Another limitation of RASS in TBI patient care is that most often the patients are going to have an RASS score of −5 due to the nature of their injury and most health care providers will grade them a −5 even with differing activities in the brain. Furthermore, a RASS score of −5 is often a goal parameter for these patients and it is likely to be over reported on the bedside chart by the nursing staff. BIS also has some limitations that require more research, such as unreliability with patient sleep state, drugs used (ketamine and nitrous oxide), hypothermia, and BIS having only been validated in non-TBI populations.^14^ Thus, there are many underlying patient cofactors that influence the accuracy of BIS, and further work into the objective measure of sedation depth needs to be explored.

Future studies should aim to continue exploring objective measures of depth of sedation with a larger multicenter study. Furthermore, RASS should be compared with objective measures of sedation (BIS) in non-TBI patients as well and probed if the relationship still exists in other circumstances when a patient is sedated. Future studies should also look at the relationship between RASS and BIS when the patient’s RASS score is between −2 and 4, to demonstrate this relationship when the patient is in a more conscious state. Future studies should trend toward an individualized momentary care and optimizing it. This pilot analysis is expected to be complementary to our parallel research line that has shown individualized depth of sedation targets in this population,^12,16,48^ and aid in sparking larger multicenter cerebral physiological studies on the subject by utilizing national and international collaborative links already existing with the Winnipeg Acute TBI Laboratory.^2,7,18,49–52^ RASS has limitations clinically in terms of reliability, and thus, an objective measure of sedation should be prioritized for further research.

## Conclusion

Through this pilot study, there appears to be limited association between RASS and BIS, and other objective measures of cerebrovascular physiology. This is highlighted by spread and substantial variability within each specific RASS value and questions the reliability of using RASS for the goal of sedation-mediated neuroprotection. Such findings are preliminary, and a larger investigation is needed to move away from a guideline-based approach and move toward a personalized approach using an objective measure of sedation for moderate/severe TBI patients in the ICU.

## Author Disclosure Statement

The authors report no conflicting interests.

## Figures and Tables

**FIG. A1.**
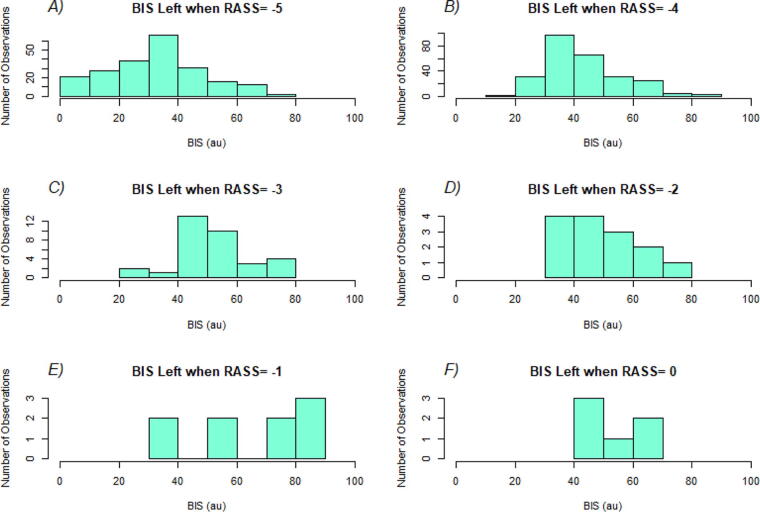
Histograms for BIS left with RASS scores of −5 to 0. BIS, bispectral index; RASS, Richmond Agitation Sedation Scale; Au, arbitrary units.

**FIG. A2.**
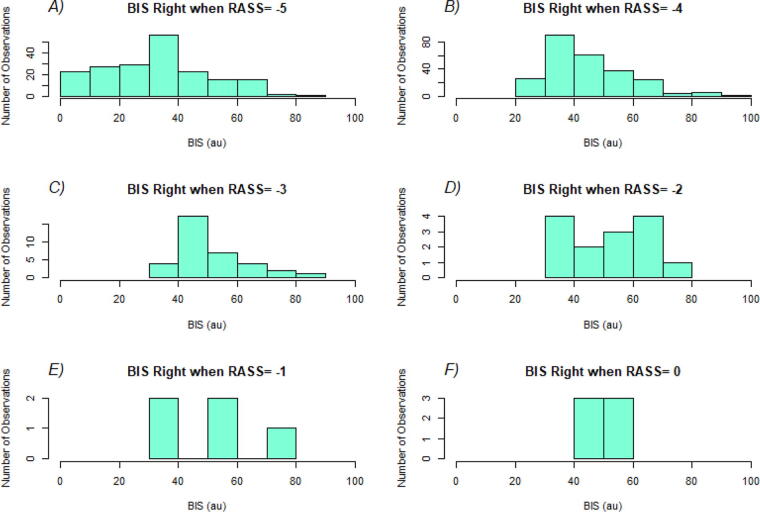
Histograms for BIS Right with RASS scores of −5 to 0. BIS, bispectral index; RASS, Richmond Agitation Sedation Scale; Au, arbitrary units.

**FIG. A3.**
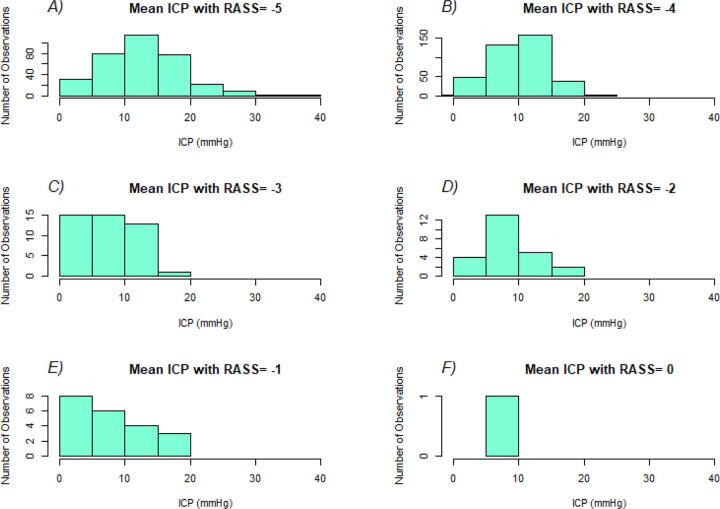
Histograms of mean ICP with RASS scores of −5 to 0. ICP, intracranial pressure; RASS, Richmond Agitation Sedation Scale; mmHg, millimeters of mercury.

**FIG. A4.**
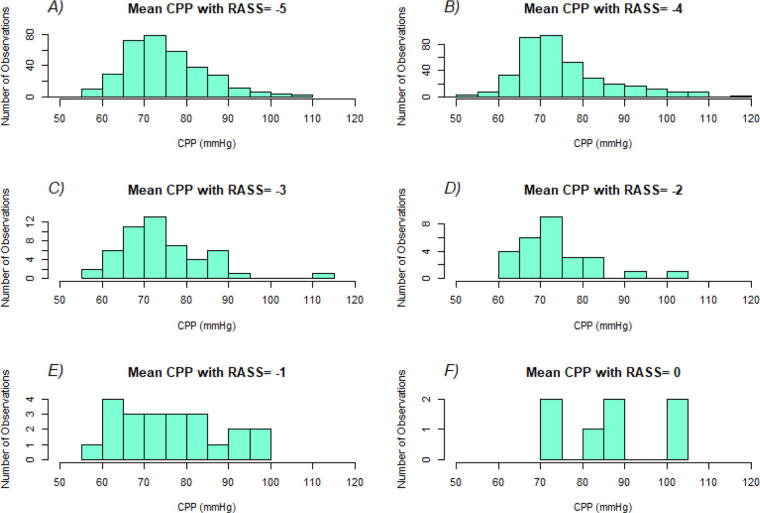
Histograms of mean CPP with RASS scores of −5 to 0. CPP, cerebral perfusion pressure; RASS, Richmond Agitation Sedation Scale; mmHg, millimeters of mercury.

**FIG. A5.**
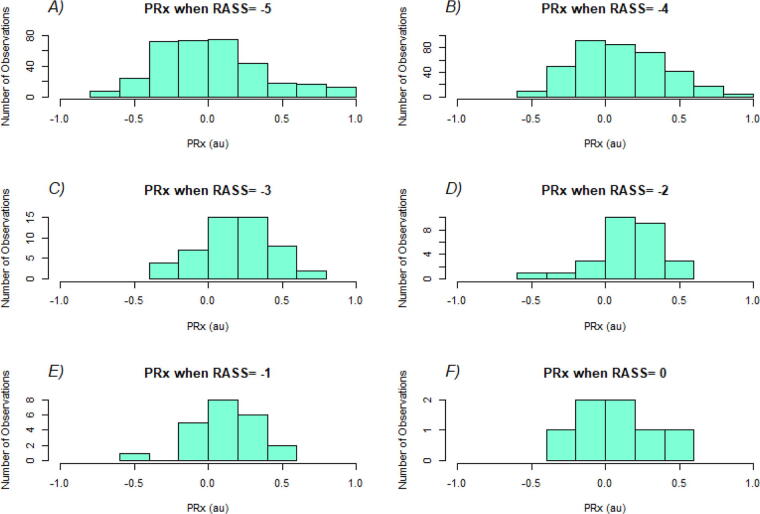
Histograms of mean PRx with RASS scores of −5 to 0. PRx, pressure reactivity index; RASS, Richmond Agitation Sedation Scale; Au, arbitrary units.

**FIG. A6.**
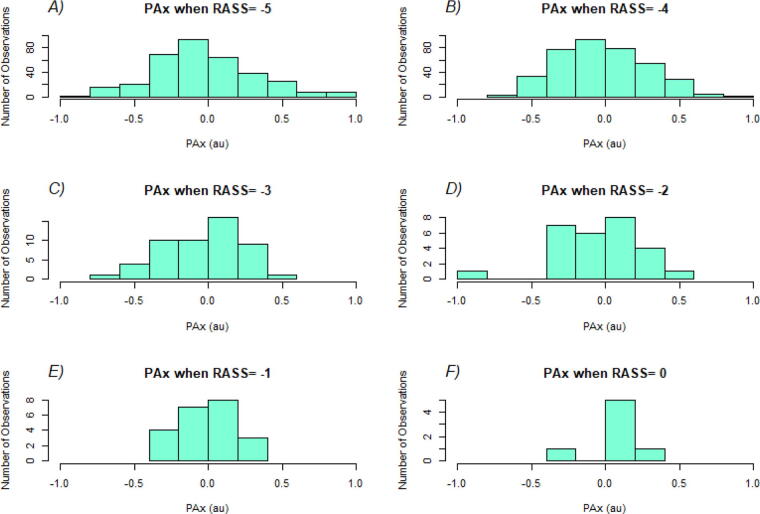
Histograms of mean PAx with RASS scores of −5 to 0. PAx, pulse amplitude index; RASS, Richmond Agitation Sedation Scale; Au, arbitrary units.

**FIG. A7.**
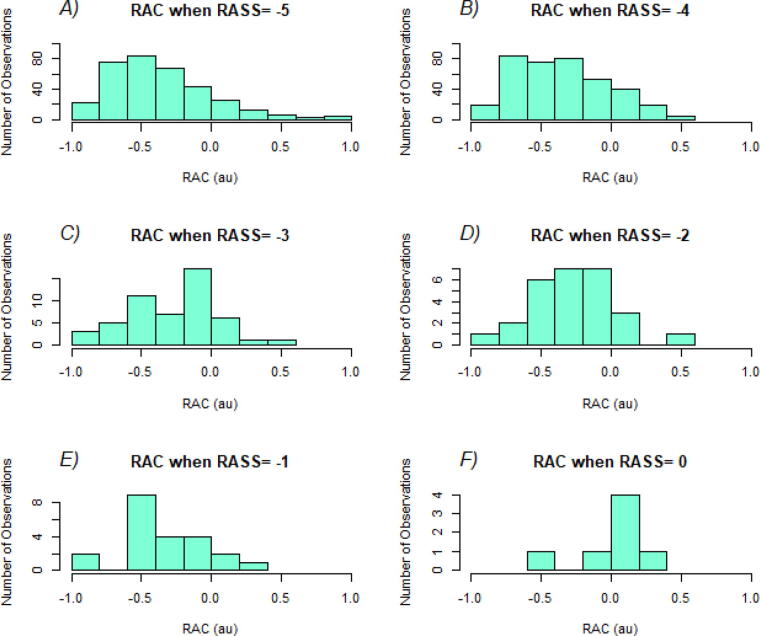
Histograms of mean RAC with RASS scores of −5 to 0. RAC, correlation between slow-waves of AMP and CPP; RASS, Richmond Agitation Sedation Scale; Au, arbitrary units.

**FIG. A8.**
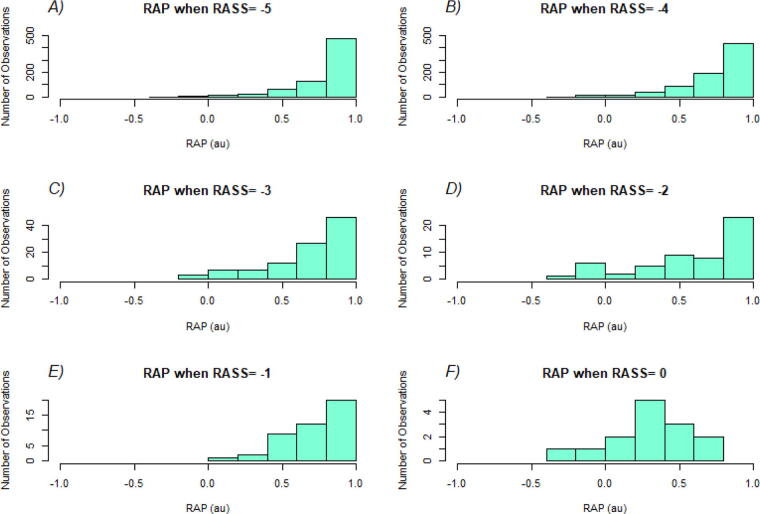
Histograms of mean RAP with RASS scores of −5 to 0. RAP, compensatory reserve; RASS, Richmond Agitation Sedation Scale; Au, arbitrary units.

**FIG. A9.**
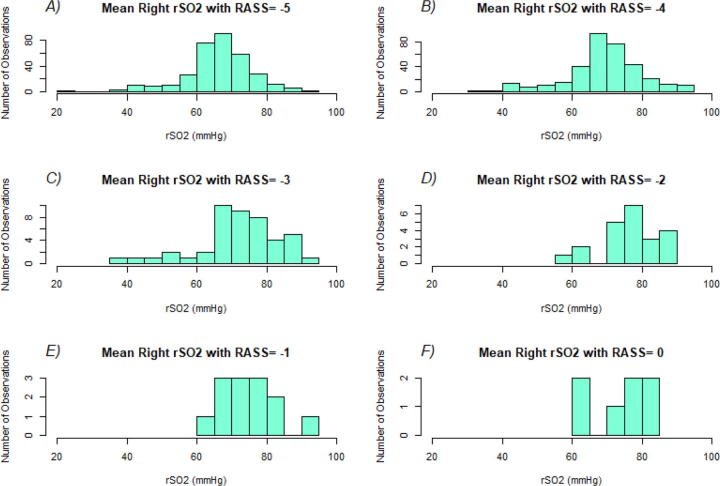
Histograms of mean rSO2 right with RASS scores of −5 to 0.rSO2, regional oxygen saturation; RASS, Richmond Agitation Sedation Scale; mmHg, millimeters of mercury.

**FIG. A10.**
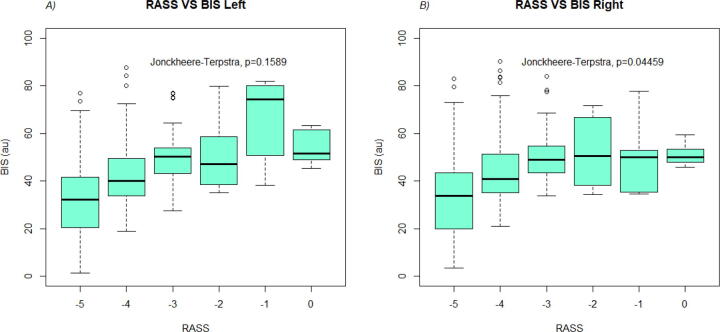
Boxplots of RASS versus BIS left and right with Jonckheere–Terpstra testing. RASS, Richmond Agitation Sedation Scale; BIS, bispectral index; Au, arbitrary units. Cerebral physiology (ICP, CPP).

**FIG. A11.**
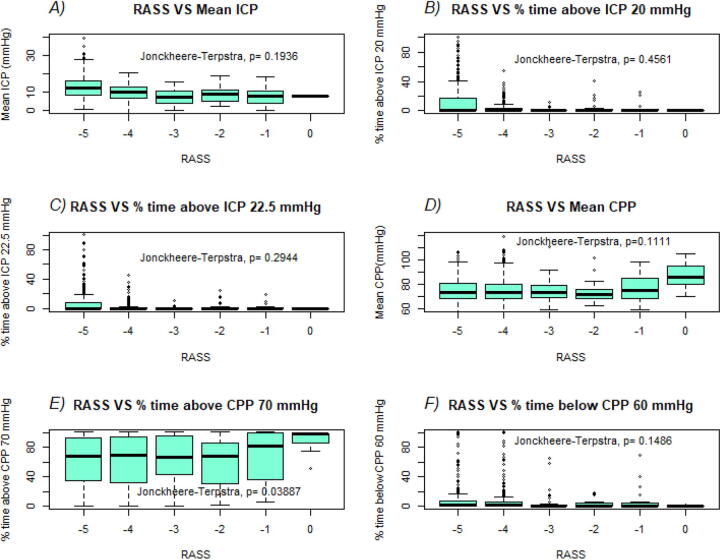
Boxplots of RASS versus ICP and CPP (mean and literature-defined thresholds) with Jonckheere–Terpstra testing. RASS, Richmond Agitation Sedation Scale; ICP, intracranial pressure; CPP, cerebral perfusion pressure; mmHg, millimeters of mercury.

**FIG. A12.**
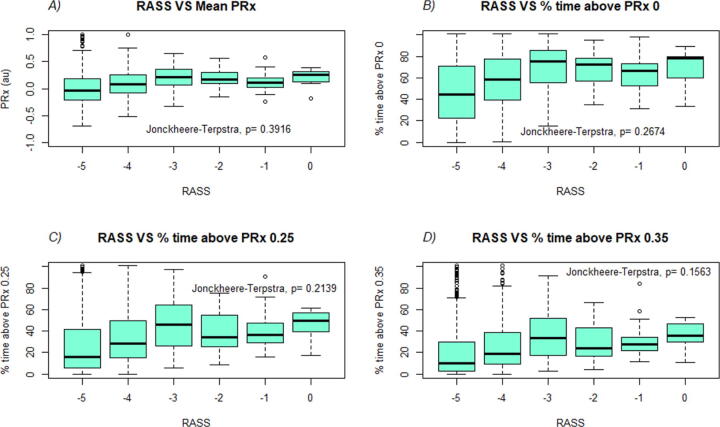
Boxplot of RASS versus PRx (mean and literature-defined thresholds) with Jonckheere–Terpstra testing. RASS, Richmond Agitation Sedation Scale; PRx, pressure reactivity index; Au, arbitrary units.

**FIG. A13.**
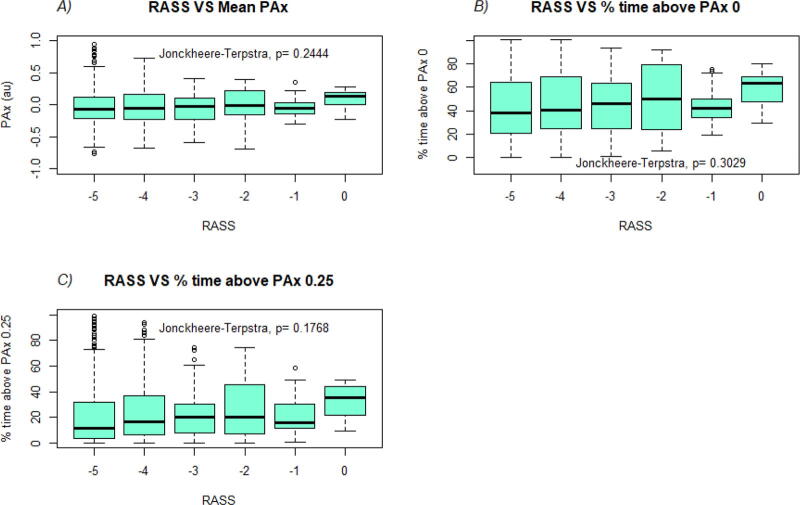
Boxplot of RASS versus PAx (mean and literature-defined thresholds) with Jonckheere–Terpstra testing. RASS, Richmond Agitation Sedation Scale; PAx, pulse amplitude index; Au, arbitrary units.

**FIG. A14.**
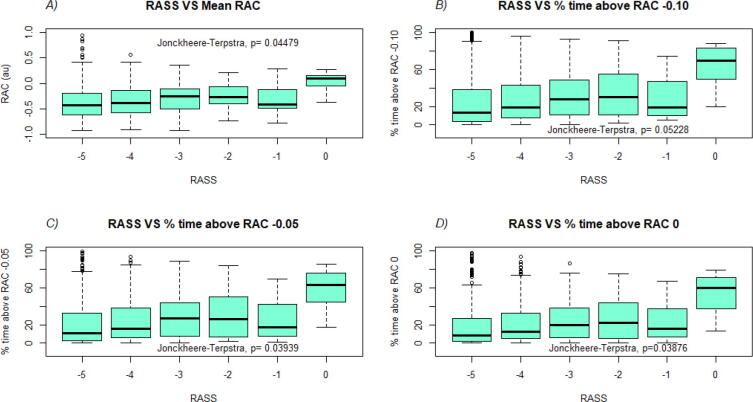
Boxplot of RASS versus RAC (mean and literature-defined thresholds) with Jonckheere–Terpstra testing. RASS, Richmond Agitation Sedation Scale; RAC, a correlation between the pulse amplitude of ICP and cerebral perfusion pressure.

**FIG. A15.**
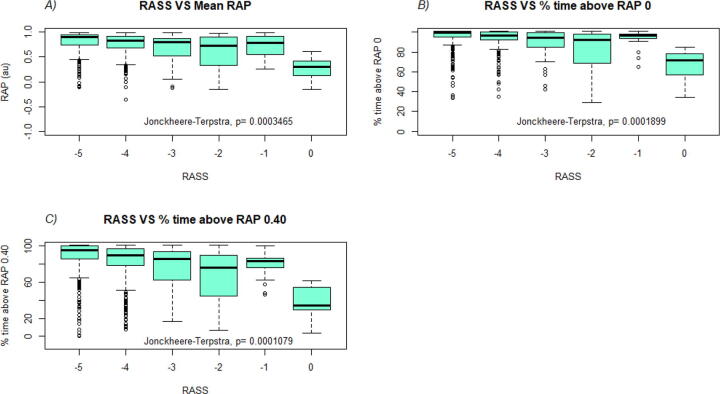
Boxplot of RASS versus RAP (mean and literature-defined thresholds) with Jonckheere–Terpstra testing. RASS, Richmond Agitation Sedation Scale; RAP, compensatory reserve; Au, arbitrary units.

**FIG. A16.**
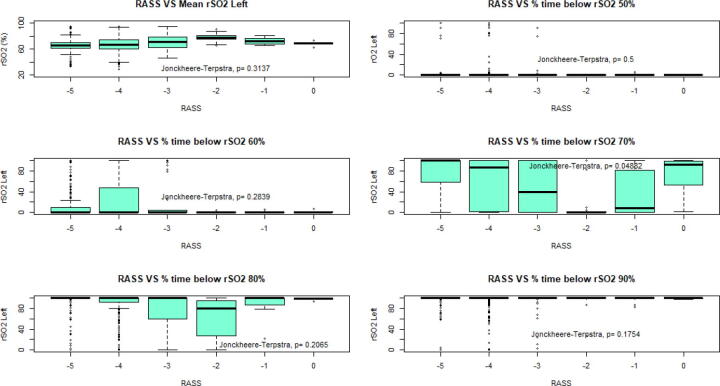
Boxplot of RASS versus mean rSO_2_ left and various rSO_2_ literature thresholds with Jonckheere–Terpstra testing. RASS, Richmond Agitation Sedation Scale; rSO_2_, regional cerebral oxygen saturation.

**FIG. A17.**
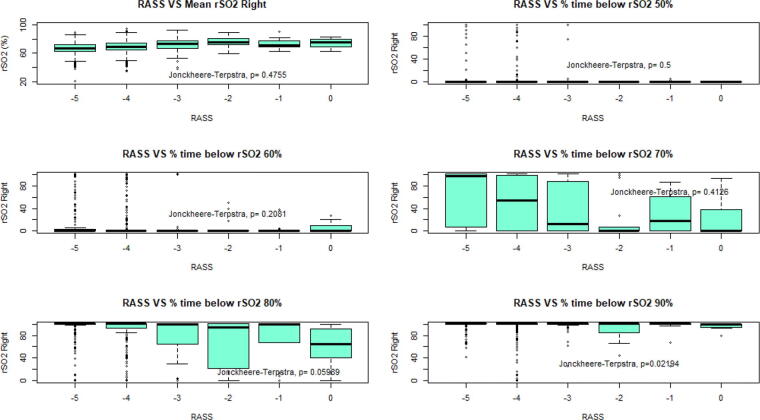
Boxplot of RASS versus mean rSO_2_ right and various rSO_2_ literature thresholds with Jonckheere–Terpstra testing. RASS, Richmond Agitation Sedation Scale; rSO_2_, regional cerebral oxygen saturation.

**FIG. A18.**
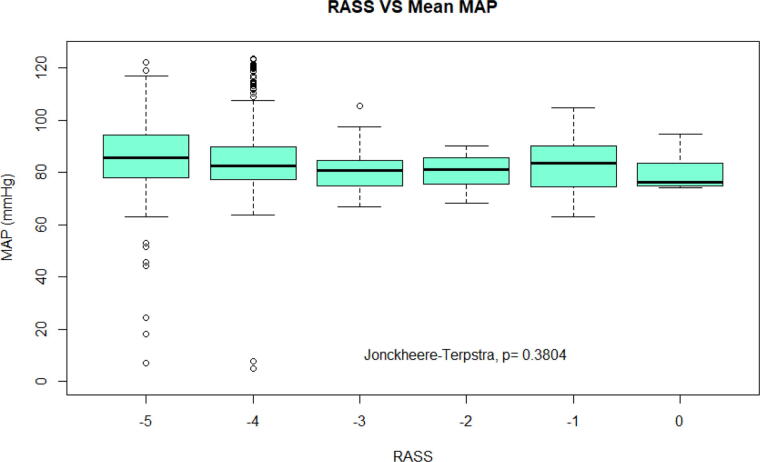
Boxplot of RASS versus MAP with Jonckheere–Terpstra testing. RASS, Richmond Agitation Sedation Scale; MAP, mean arterial pressure; mmHg, millimeters of mercury.

**FIG. A19.**
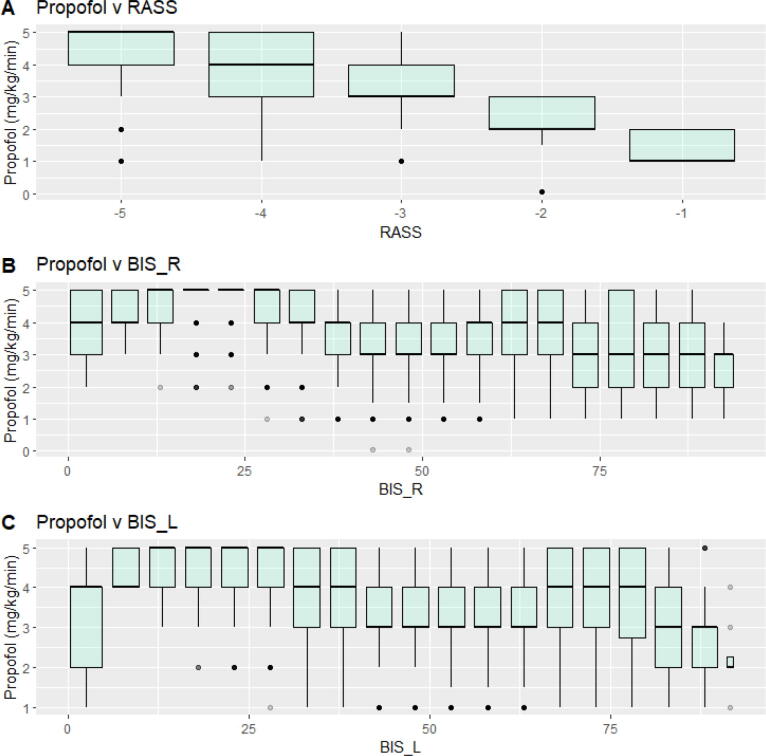
Propofol and RASS/BIS. RASS, Richmond Agitation Sedation Scale; h, hour; kg, kilogram; mg, milligram; min, minute; BIS, bispectral index; µg, microgram; Panel A demonstrates propofol and RASS, Panels B/C are propofol of BIS left/right side using boxplots.

**FIG. A20.**
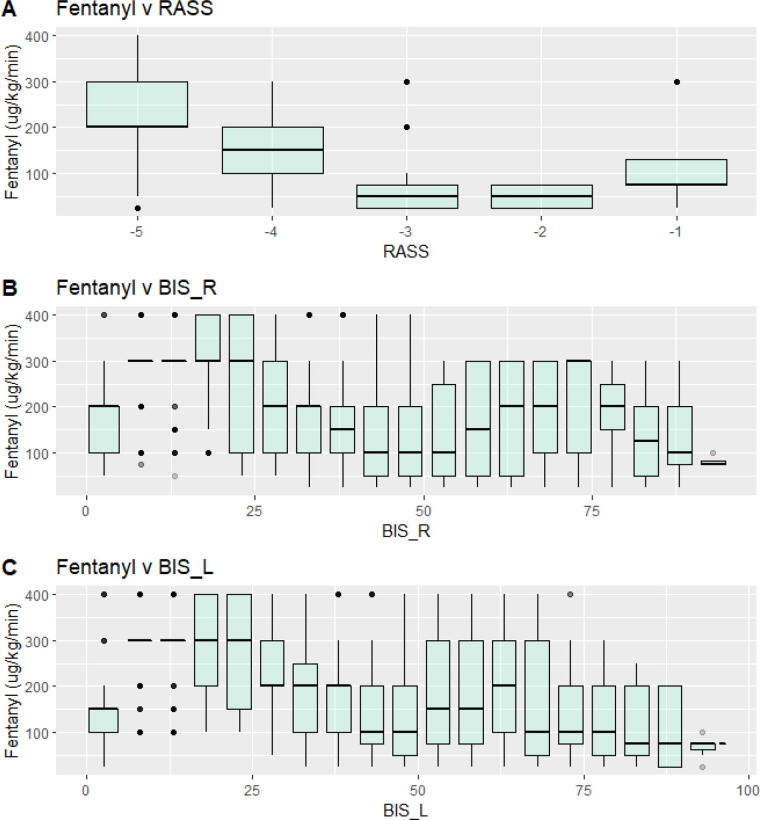
Fentanyl and RASS/BIS. RASS, Richmond Agitation Sedation Scale; h, hour; kg, kilogram; mg, milligram; min, minute; BIS, bispectral index; µg, microgram; Panel A demonstrates fentanyl and RASS, Panels B/C are propofol of BIS left/right side using boxplots.

**FIG. A21.**
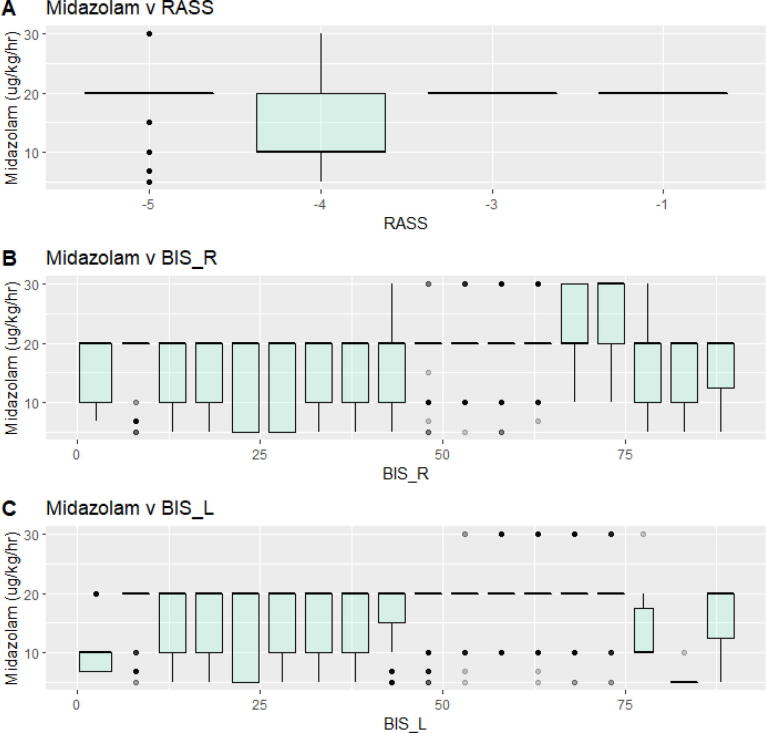
Midazolam and RASS/BIS. RASS, Richmond Agitation Sedation Scale; h, hour; kg, kilogram; mg, milligram; min, min; BIS, bispectral index; µg, microgram; Panel A demonstrates midazolam and RASS, Panels B/C are propofol of BIS left/right side using boxplots.

## Abbreviations Used

ABP: arterial blood pressure
AMP: pulse amplitude of intracranial pressure
Au: arbitrary units
BIS: bispectral index
BIS_L: bispectral index left
BIS_R: bispectral index right
BTF: Brain Trauma Foundation
CPP: cerebral perfusion pressure
EEG: electroencephalogram
GCS: Glasgow Coma Scale
ICU: intensive care unit
IQR: interquartile range
ICM+: Intensive Care Monitoring “Plus”
ICP: intracranial pressure
MAP: mean arterial pressure
NIRS: near-infrared spectroscopy
PAx: pulse amplitude index
PRx: pressure reactivity index
pCO_2_: partial pressure of carbon dioxide
pO_2_: partial pressure of oxygen
RAC: correlation between pulse amplitude of intracranial pressure and cerebral perfusion pressure
RAP: compensatory reserve
RASS: Richmond Agitation Sedation Scale
rSO_2_: regional cerebral oxygen saturation
TBI: traumatic brain injury

## Appendix 4. Boxplots of Cerebral Physiology Versus BIS

The Jonckheere–Terpstra test was not significant for all comparisons. RASS, Richmond Agitation Sedation Scale; BIS, bispectral index; Au, arbitrary units; ICP, intracranial pressure; CPP, cerebral perfusion pressure; mmHg, millimeters of mercury, PRx, pressure reactivity index; PAx, pulse amplitude index; RAC, a correlation between the pulse amplitude of ICP and CPP; RAP, compensatory reserve; rSO_2_, regional cerebral oxygen saturation.
